# The Effect of microRNA on the Production of Recombinant Protein in CHO Cells and its Mechanism

**DOI:** 10.3389/fbioe.2022.832065

**Published:** 2022-03-21

**Authors:** Hui-Ning Liu, Wei-Hua Dong, Yan Lin, Zhao-Hui Zhang, Tian-Yun Wang

**Affiliations:** ^1^ The First Affiliated Hospital of Xinxiang Medical University, Xinxiang, China; ^2^ International Joint Research Laboratory for Recombinant Pharmaceutical Protein Expression System of Henan, Xinxiang, China; ^3^ Department of Biochemistry and Molecular Biology, Basic Medical School, Xinxiang Medical University, Xinxiang, China

**Keywords:** CHO cells, miRNA, recombinant protein, cell engineering, protein quality

## Abstract

Recombinant protein production by mammalian cells is the initial step in the manufacture of many therapeutic proteins. Chinese hamster ovary (CHO) cells are the most common host system to produce recombinant therapeutic proteins (RTPs). However, it is still challenging to maintain high productivity ensuring the good quality of RTPs produced by CHO cells. MicroRNAs(miRNAs) are short regulatory non-coding RNAs that can regulate cellular behavior and complex phenotypes. It has been found that miRNAs can enhance the expression level of recombinant proteins in CHO cells by promoting proliferation, resisting apoptosis, and regulating metabolism. miRNAs also can affect the quality of RTPs. In this review, we will discuss the effect and mechanism of miRNA on the expression level and quality of recombinant proteins in CHO cells.

## Introduction

Recombinant therapeutic proteins (RTPs) are produced in prokaryotic and eukaryotic cells. Recombinant biologic drugs have many advantages that traditional small molecule drugs do not have. Post-translational modifications (PTMs) of the host system can directly affect the effectiveness and immunogenicity of RTPs. Mammalian cells are often employed for manufacture of RTPs as they are capable of PTMs which make the resulting materials suitable for clinical use (Nettleship et al., 2015; Stepanenko and Dmitrenko, 2015; Dumont et al., 2016; Bürgin et al., 2020). Among them, nearly 70% of RTPs approved by the FDA are manufactured from Chinese hamster ovary (CHO) cells (Kim et al., 2012).

Compared with other expression systems, CHO cells have better adaption to high-density suspension culture, low sensitivity to human virus infection, PTMs similar to human cells and the incidence of immune reaction is low (Hackl et al., 2012; Jadhav et al., 2013; Lai et al., 2013). In addition, many CHO gene amplification systems have been established, such as the gene amplification system mediated by dihydrofolate reductase or glutamine synthetase used for screening to obtain higher recombinant protein production (Kim et al., 2012); Exogenous genes can be stably integrated into CHO cells; CHO cells are fibroblasts and hardly secrete endogenous proteins, so it is very beneficial for the separation and purification of recombinant proteins (Li et al., 2018). Glycosylation is one of the critical quality attributes to produce recombinant proteins by CHO cells. The glycosylation patterns of recombinant proteins directly affect these drugs’ clinical efficacy and safety by modulating their stability, biological activity, circulatory half-life, and pharmacokinetics. Numerous strategies have been investigated to control the glycan profiles of recombinant proteins including glycoengineering on the glycosylation pathways and metabolite supplementation to the culture media. Glycoengineering involves the modulation of glycogen expression in CHO cells, which includes overexpression of sialyltransferase to enhance terminal sialylation and N-acetylglucosaminyltransferases to improve branching (Wang Q. et al., 2019). Overexpression of *ß*-1,4-galactosyltransferase (β4GALT1) enhances the glycosylation of monoclonal antibodies (Dekkers et al., 2016). RNA interference is used to inhibit the expression of the *α*-1,6-fucosyltransferase (FUT8) gene to reduce fucosylation (Mori et al., 2004). Fructosylation is achieved by knocking out FUT8 or the enzyme responsible for guanine diphosphate-fucose biosynthesis (Chang et al., 2019). Although the yields of RTPs have been increased through the efforts in the past 20 years, it is still challenging to maintain the high expression level and quality of RTPs produced by CHO cells. It has been reported that the performance of CHO cells can be improved by gene editing technology, such as zinc finger nucleases and CRISPR/Cas9 system, thus increasing recombinant protein expression and quality (Krämer et al., 2010). Transgene can be also increased by optimizing the design of the expression vector or overexpressing the anti-apoptotic gene (Kim et al., 2011; Hackl et al., 2012). DNA methylation can be reduced through establishment of the DNA methyltransferase-deficient (Dnmy3a-deficient) CHO cells to improve the expression level and stability of exogenous genes (Jia et al., 2018; Wang X. Y. et al., 2019). miRNAs have recently been used to improve the phenotype of CHO cells related to yield and quality. There is growing evidence that overexpression or inhibition of miRNAs can increase the level of recombinant protein expression produced by CHO cells. These small endogenous RNAs can regulate cellular pathways, and overexpression in cells will not compete with the host cell’s translation mechanism (Meleady et al., 2012).

MicroRNAs (miRNAs) are regulatory non-coding RNAs with a fragment length of approximately 23 nucleotides (Cora' et al., 2017), which are naturally encoded in the genome. miRNAs are transcribed by RNA polymerase II in the nucleus to form a primary transcript with a hairpin structure, that is, pri-miRNA, which contain one or a few stem-loop structures composed of approximately 70 nucleotides each. In the nucleus, pri-miRNAs are cleaved into a stem-loop structure called precursor miRNAs (pre-miRNAs) by Drosha. After export of pre-miRNAs to the cytoplasm by Exportin-5, they are cleaved near the loop into small double-stranded RNAs (dsRNAs) by Dicer. The guide strand of the dsRNA with the Argonaute2 protein form an RNA-induced silencing complex (RISC). miRNA molecules generally bind to the 3′-untranslated region(3′-UTR) of target mRNA in an imperfect complementary manner and regulate the expression of recombinant proteins by mRNA destabilization and translational repression, which belongs to the post-transcriptional regulation level. And one miRNA can bind multiple target mRNAs (Fischer et al., 2015a; Saliminejad et al., 2019; Leroux et al., 2021). Overexpression of miRNAs is mainly done by constructing efficient expression vectors or synthesizing mimics. Three general approaches are used to study miRNA loss of function, including genetic knockouts, antisense oligonucleotide inhibitors, and miRNA sponge (Ebert and Sharp, 2010; Jadhav et al., 2013). This paper reviews the progress of research on the effects of miRNAs on the expression level and quality of recombinant proteins in CHO cells.

## Effect of miRNA on the Production of Recombinant Protein

Currently, in CHO cells, several studies have demonstrated that transient or stable overexpression of miRNAs can increase the yield of recombinant proteins in CHO cells without affecting the quality of recombinant proteins.

### miRNAs Improve the Yields of Recombinant Protein

Many studies have proved that overexpression of miRNA can increase the yield of recombinant protein produced by CHO cells. Stable overexpression of miR-17 can double the specific productivity of CHO cells expressing EpoFc and triple the titer (Jadhav et al., 2014). The stable overexpression of miR-106b increased the titer of IgG antibody produced by CHO cells by 0.66 times (Xu et al., 2019). Overexpression of the miR-30 family (including miR-30a, miR-30b, miR-30c, miR-30d, and miR-30e) promoted the production of secreted alkaline phosphatase (SEAP) in CHO cells by approximately two-fold (Fischer et al., 2014). Stable overexpression of miR-2861 downregulates histone deacetylase 5 (HDAC) in CHO cells and increases the cellular productivity of various recombinant proteins (SEAP, mAb) produced by different CHO cell lines (Fischer et al., 2015b). Stable overexpression of miR-17, miR-19b, miR-20a, and miR-92a in low yielding CHO K1-mAb clone SH31 and high yielding monoclonal antibody CHO K1-mAb clone SH87 promoted monoclonal antibody specific productivity by 24–34% and titer by 21–31%, and then the highest yielding isolated from these stable transfection pools clones with the 1.3∼1.4-fold increase in potency compared to the parental clones (Loh et al., 2014). The stable overexpression of hsa-miR-557 and hsa-miR-1287 in CHO cells can significantly increase the production of IgG and recombinant human serum albumin by more than 1.5 times, Co-expression of miR-557 and miR-1287 resulted in a significant increase in IgG concentration of approximately 1.3-fold compared to negative control and parental cells (Strotbek et al., 2013). The introduction of miR-483 mimics will also significantly increase the production of antibodies in CHO cells (Emmerling et al., 2016). Overexpression of miR-31* can increase IgG production and cell-specific productivity (Martinez-Lopez et al., 2021). Overexpression of miR-219-1-3p and miR-3074-5p can increase the production of antibodies in high-yielding CHO cell lines. Among them, the yield of overexpression of miR-3074-5P is about 8 times higher than that of the negative control. In the low-yielding CHO cell expression line, overexpression of miR-136-3p increased the antibody production by more than 12 times. In addition, in the CEVEC’s amniocyte production cell line, the antibody concentrations of overexpressing miR-136, miR-3074, and miR-219 were 1.13, 1.06, and 1.15 times that of the negative control, respectively (Weis et al., 2018). When miR-574-3p is overexpressed in CHO cells, it can increase the titer of erythropoietin and etanercept from 1.3 to 2 times (Švab et al., 2021). Plasma cells (PCs), as part of the adaptive immune system, are naturally specialized in their structure, function, and cell signaling pathways for stable and high-level antibody production. Raab et al. screened 14 miRNAs from a subset of plasma cell-specific miRNAs for improving parameters related to biological processes in CHO cells. Two different cell lines expressing different monoclonals, CHO-DG44-mAb1 and CHO-K1-mAb2, were also selected. miR-182-5p, miR-421-4p, miR-130b-3p, miR-183-5p, miR-1839-5p miR-374b-5p and miR-1839-3p, increased the specific productivity of CHO-DG44-mAb1 cells by 15–23%, in addition, miR-130b-3p, miR-374B-5p and miR-1839-3p increased the antibody titer by 14–19%. In CHO-K1-mAb2 cells, miR-183-5p, miR-17-3p, miR-138-5p, miR-342-5p, and miR-491-5p resulted in a significant increase in unit yield by 13–50%, while miR-711, miR-484, and miR-425-5p also exhibited growth-promoting properties, contributing to an antibody yield increased by 16–26% (Raab et al., 2021).

However, the overexpression of some miRNAs will hurt the expression of recombinant proteins, and the production of recombinant proteins can be improved by inhibiting these miRNAs. Sanchez et al. used miRNA “sponge” technology to inhibit effective endogenous miR-7 and found that in fed-batch culture, inhibiting miR-7 would almost double the production of SEAP in CHO cells (Sanchez et al., 2014). miR-7 sponge was stably transfected into the DP12 cell line, the density of viable cells increased by 65%, and the IgG secreted by the cells increased by 3 times (Coleman et al., 2019). By stably inhibiting mmu-miR-466h-5p, Druz et al. found that compared with negative control cells and parental CHO cells, the expression of SEAP in CHO cells resistant to mmu-miR-466h-5p increased by 43%, and the cell specific productivity increased by 11% (Druz et al., 2013). Studies have shown that by stably consuming miR-23, the production of SEAP in CHO cells can be increased by three times without affecting growth (Kelly et al., 2015). CRISPR/Cas9-mediated miR-744 gene knockout significantly increased the antibody titer of the CHO cell line to 190–311 mg/L compared to the control 156 mg/L by up-regulating the miR-744 target gene (Raab et al., 2019).

### miRNAs Promote the Expression of “Difficult-to-Express” Protein

Large-scale production of recombinant proteins in mammalian cells may be affected by many potential limiting steps or “bottlenecks” in protein expression pathways. Recombinant proteins derived from many mammalian cells are secreted. The balance between different processes, related factors and protein quality control mechanisms will affect the rate of protein secretion. Due to the complexity of mammalian expression systems, limitations may give rise to unpredictability in the production of certain recombinant proteins. The so-called “difficult to express” proteins are affected by potential limiting steps or “bottlenecks” that prevent the secretion of a sufficient number of recombinant proteins (Hussain et al., 2017; Kaneyoshi et al., 2019). Studies have found that the stable overexpression of miR-557 increases the potency of hard-to-express monoclonal antibodies twofold. In addition, the clones expressing miR-557 showed better overall process performance, higher mAb titer, higher growth value, longer survival rate, and lower cell-specific lactate production. The presence of miR-557 significantly enhanced each process step during cell line development in a product-independent manner. (Fischer et al., 2017). Schoellhorn et al. selected a CHO cell line that produced a difficult-to- express mAb at <200 mg*L-1 in an 8-days batch cultivation. Stable overexpression of miRNA-143 in the CHO-mAb cell line increased the yield of difficult-to- express mAb by downregulating MAPK7 (Schoellhorn et al., 2017). Transient inhibition of CHO endogenous miR-23a/miR-377 resulted in approximately 15–21% specific activity of hard-to-express recombinant lysosomal sulfatase (Amadi et al., 2020). The miRNAs that can enhance the expression of recombinant proteins in CHO cells are reviewed above, and a table is presented (table1).

**TABLE 1:** The effect of miRNAs on expression of recombinant protein in CHO cells.

Type	Protein of interest	Results	Target gene	References
miR-17	EpoFc	Two-fold increased in productivity	—	Jadhav et al. (2014)
miR-106b	IgG	Two-fold increased in productivity	CYLD NF-κB and Wnt/β-catenin	Xu et al. (2019)
miR-30 family	SEAP	Double the output	Skp2	Fischer et al. (2015c)
miR-2861	mAb	Increased	HDAC5	Fischer et al. (2015b)
miR-17/-92a/20a	mAb	Specific productivity increased by 24–34%, titer increased by 21–31%I	—	Loh et al. (2014)
miR-557	IgG and recombinant human serum albumin	Increased more than 1.5 times in IgG and human serum albumin production	—	Strotbek et al. (2013)
miR-1287				
co-expression of miR-557 and miR-1287	IgG	IgG concentration was significantly increased about 1.3-fold	—	Strotbek et al. (2013)
miR-483	mAb	Increase antibody production	—	Emmerling et al. (2016)
miR31*	IgG	Improve IgG production and cell specific productivity	—	Martinez-Lopez et al. (2021)
miR-3074-5p	mAb	Increased 8 times	—	Weis et al. (2018)
miR-136-3p	mAb	Increased the product yield by more than 12 times	—	Weis et al. (2018)
miRNA-574-3p	EPO and ETN	The titers of EPO and ETN increased from 1.3 to 2 times	P300	Švab et al. (2021)
miR-182-5p, miR-421-4p, miR-130b-3p, miR-183-5p, miR-1839-5p, miR-374b-5p miR-1839-3p	mAb	Increased the specific productivity by 15–23%	SETD2 Kmt1a Whsc1l Kmt5b Kmt2d	Raab et al. (2021)
miR-183-5p, miR-17-3p, miR-138- 5p, miR-342-5p, and miR-491-5p	mAb	Increased specific productivity of 13–50%	CPT1A Scd2 Acadl Oxsm	Raab et al. (2021)
miR-7	SEAP	Increased nearly two times	—	Sanchez et al. (2014)
miR-7	IgG	Increased production threefold	Skp2 Psme3 SG2NA Ezrin BMS1	Coleman et al. (2019)
miR-466h-5p	SEAP	Increased by 43%, cell-specific productivity increased by 11%	bcl2l2 Birc6 dad1 stat5a smo	Druz et al. (2013)
miR-23	SEAP	Increased three times	—	Kelly et al. (2015)
miR-744	mAb	Increased to 190–311 mg/L compared to the control 156 mg/L	—	Raab et al. (2019)
miRNA-577	difficult-to-express proteins	Increased two-fold	—	Fischer et al. (2017)
miR-143	difficult-to-express proteins	Increased the production	—	Schoellhorn et al. (2017)
miR-23a	Recombinant Sulfatase	Increased specific activity by about 15–21%	—	Amadi et al. (2020)
miR-377				

### miRNAs Affect the Quality of Recombinant Protein

In addition to the yield, the aggregation and glycosylation characteristics of the RTPs are also crucial because glycosylation is one of the important quality attributes of recombinant proteins and they determine the functionality, efficacy, safety, and *in vivo* half-life of the protein (Gupta and Shukla, 2018). To determine whether the increase in yield caused by overexpression of miRNA was accompanied by changes in the quality of the recombinant protein, Loh et al. isolated a high-yield clone from each cell pool stably transfected with miR-17, miR-19b, miR-20a, and miR-92a for aggregation and N-glycosylation. The results showed that the overexpression of miRNA had no effect on IgG aggregation and N-glycosylation, and all purified IgG samples showed low aggregation of less than 1%. And it is consistent with the distribution pattern of recombinant human IgG polysaccharides produced by normal CHO cells (Loh et al., 2014). After Fischer and others stably overexpressed miR-2861 in CHO cells producing monoclonal antibodies, the analysis of the culture supernatant and the N-glycosyl chain of monoclonal antibodies showed that the formation of monoclonal antibody aggregation and the N-glycosylation map of antibody secretion was not affected, which also indicated that the overexpression of miR-2861 did not affect the quality of antibodies (Fischer et al., 2015b). In CHO-IgG cells, Xu et al. found that stable overexpression of miR-106b also did not affect antibody quality (Xu et al., 2019). Fischer et al. found that overexpression of miR-557 had no negative effect on N-linked glycosylation, mAb aggregation/fragmentation, and antibody chain purity and integrity without negative effects (Fischer et al., 2017). Fischer et al. showed that in CHO cells, a single point mutation in the promoter region of the cgr-miR-111 host gene sidekick cell adhesion molecule 1 (SDK1) caused deletion of miR-111. The resulting silencing of SDK1 and miR-111 leads to deregulation of (CMP)-N-acetylneuraminic acid hydroxylase (CMAH) expression and ultimately to increased N-glycolylneuraminic acid (NGNA) sialylation of recombinant mAb (Fischer et al., 2021). At present, studies on the effects of miRNA on recombinant protein glycosylation in CHO cells are scarce and need to be further explored.

## Mechanism of miRNA Affecting the Expression of Recombinant Protein

miRNAs can bind to multiple target mRNAs and regulate the expression of related proteins. The best way to understand the function of miRNAs is to identify the genes that it regulates. Most of the research on miRNA mechanisms has focused on two main areas: elucidating miRNA-mediated post-transcriptional gene regulation mechanisms and identifying the targets of miRNAs. This has been achieved by using well-established methods such as gene expression microarrays, next-generation sequencing, computational prediction of miRNA targets, and mass spectrometry-based proteomics approaches. Specific miRNAs have specific target genes, which depend on their sequence. Therefore, different miRNAs affect cells and their properties in different ways (Thomas et al., 2010; Huang et al., 2013). Studies have shown that miRNAs can alter the cell cycle to affect cell density, inhibit apoptosis to extend cell lifespan, target mitochondrial metabolism to improve energy flux, and otherwise influence protein expression, leading to a high-yield phenotypes.

### miRNAs Affect Cell Proliferation and Apoptosis

Manipulating miRNA levels in CHO cells can increase product yield by affecting cell proliferation and apoptosis. Transient overexpression of miR-17 increases the rate of cell proliferation without negatively affecting specific productivity (Jadhav et al., 2012). The stable overexpression of miR-106b can also significantly promote cell proliferation, improve CHO cell viability and antibody production (Xu et al., 2019). Overexpression of miR-574-3p induces the reduction of CHO-ETN cell apoptosis. The cell cycle analysis of CHO-ETN cells stably expressing miR-574-3p by flow cytometry showed that the number of cells in the G0/G1 phase was less than the control cells after four days of culture. miR-574-3p shows anti-apoptotic effect (Švab et al., 2021). Up-regulation of miR-7 by targeting the key regulators’ ubiquitin E3 ligase S-phase kinase-associated protein 2 (Skp2) and Proteasome activator complex subunit 3(Psme3) of the transition from G1 to S phase of the cell cycle induces G1 phase halt but does not promote cell apoptosis (Sanchez et al., 2013). In the early culture of CHO DP12 cells, inhibition of miR-7 by miRNA “sponge technology” can up-regulate Akt pathway-related proteins striatal protein-3 (SG2NA) and Ezrin, thereby increasing cell density and promoting cell growth (Coleman et al., 2019). Druz et al. determined the changes in miRNA expression in CHO cells undergoing apoptosis induced by exposing the cells to nutrient-depleted media. In nutrient-depleted media, mmu-miR-466 h expression was up-regulated in CHO cells. Inhibition of mmumiR-466 h increased the mRNA levels of bcl2l2, Birc6, dad1, stat5a and smo anti-apoptotic genes 4∼23 fold, suggesting that mmu-miR-466 h showed pro-apoptotic effect and regulated apoptotic pathways in mammalian cells (Druz et al., 2011). Muluhngwi et al. observed that 4-hydroxytamoxifen (4-OHT) increased the transcription of pri-miR-29b-1 and pri-miR-29a in CHO-K1 cells by activating the endogenous estrogen receptor (ERA). aberrant expression of miR-29b-1 and miR-29a reduced CHO-K1 cell proliferation, colony formation, and cell invasion. In addition, increased miR-29b-1/a expression reduced mRNA and protein levels in Disher (Muluhngwi et al., 2017).

### miRNAs Regulate Gene Expression in the Post-transcriptional Levels

In CHO-K1 cells, miR-106b overexpression can directly target the 3′UTR of deubiquitinating CYLD and inhibit the mRNA and protein levels of CYLD at the post-transcriptional level, thereby enhancing antibody expression. Stable overexpression of miR-106b enhanced the expression of *ß*-catenin and Phosph-p65 in CHO-Ig G cells. Thus, stable overexpression of miR-106b also triggered the activation of NF-κB and Wnt/β-catenin signaling pathways (Xu et al., 2019). By stably overexpressing miR-2861, down-regulating the expression of histone HDAC5 in CHO cells after transcription will increase the production of antibodies. In mice, miR-2861 has been reported to directly regulate HDAC5 expression by binding to miRNA targets in the coding sequence of HDAC5 transcripts. Fischer et al. compared mouse miR-2861 targets with cgr-HDAC5 mRNA sequences. They found that the mouse miR-2861 target was also fully conserved in CHO cells. This demonstrated that miRNA-2861 functions by binding to the mRNA sequence of HDAC5 in CHO cells (Fischer et al., 2015b). The main effector protein of miR-574-3p is p300, which is the main acetyltransferase of p53. It acetylates p53 to stabilize p53, which inhibits CMV promoter activity and interferes with basic transcription. When miR-574-3p is overexpressed, down-regulation of mRNA levels and protein levels of p300, which reduces the acetylation, destabilization, and degradation of p53 eliminates the inhibition of the CMV promoter and increases the expression of recombinant protein (Švab et al., 2021).

### miRNAs Regulate Gene Expression at the Translation Level

Regarding the effect of miRNAs at the translational level on recombinant protein expression in CHO cells, a related study to date is the 15-fold increase in expression of ribosomal assembly proteins that play a role in ribosome biogenesis following inhibition of miRNA-7 by sponge technology in late CHO DP12 cell cultures compared to negative controls. BMS1 may be a target of miR-7 which when elevated could improve productivity by translational enhancement within ribosomes. BMS1 has not been reported in CHO cells, and this theory lays the groundwork for further studies on the specific engineering of BMS1 in CHO cells (Coleman et al., 2019).

### miRNAs Affect Cell Metabolism

**FIGURE 1:**
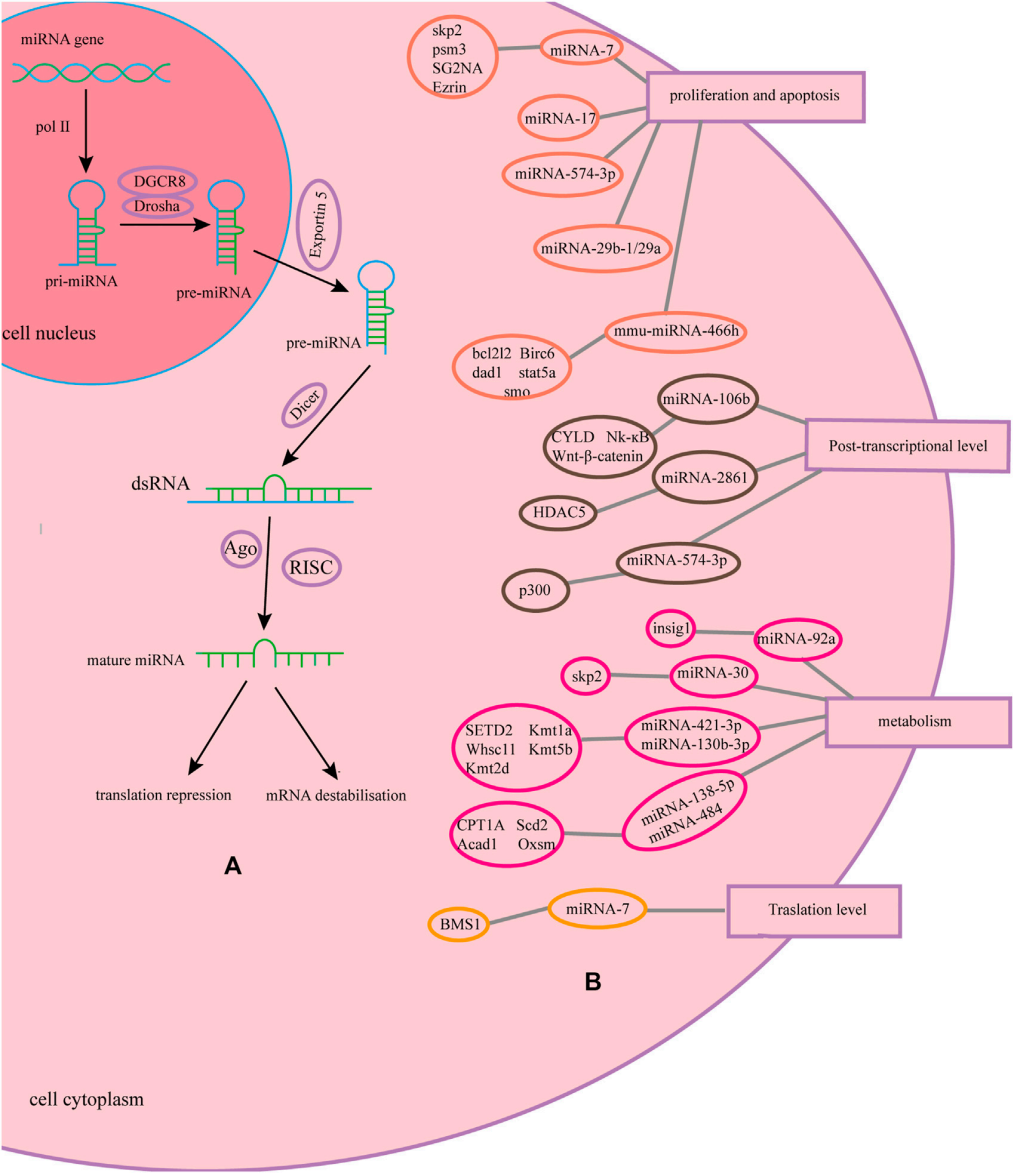
Biological process and function of miRNA and its mechanism. **(A)** Biological process of miRNA and its mechanism. **(B)** The mechanisms of miRNAs on recombinant protein in CHO cells.

The miR-30 family directly targets the Skp2 and participates in the regulation of the ubiquitin pathway of CHO cells. Overexpression of the miR-30 family will result in a significant down-regulation of Skp2, thereby increasing the protein production of CHO cells (Fischer et al., 2015c). miR-92a affects cholesterol metabolism by inhibiting the cholesterol biosynthesis regulator insig1, leading to an increase in intracellular cholesterol levels and Golgi volume, thereby increasing product yield (Loh et al., 2017). miR-31* improves the unit productivity of CHO cells by promoting oxidative phosphorylation in mitochondria. miR-23 can increase the activity of mitochondria without affecting growth (Švab et al., 2021). Inhibition of endogenous miR-378-3p in CHO-DP12 cells can increase the abundance of ubiquitin carboxyl-terminal hydrolase 14 (USP14) and increase the peak density of viable cells by 59%, but the specific cell productivity has not been improved (Costello et al., 2018). The latest study by Raab et al. found that overexpression of miR-421-3p and miR-130b-3p in CHO-DG44-mAb1 can regulate the SETD2, Kmt1a, Whsc1l, Kmt5b, and Kmt2d genes in the lysine degradation pathway. Overexpression of miR-138-5p and miR-484 in Cho-K1-mAb2 can make CPT1A, Scd2, Acadl, and Oxsm genes in the fatty acid metabolism pathway 8, 115, 1.3, and 1.1 times higher, respectively (Raab et al., 2021). We have made a summary of the mechanism of miRNA in the article with a graph, as shown in Figure 1.

## Summary and Prospect

At present, many studies have proved that the application of miRNA can affect the expression of CHO cell recombinant protein from proliferation, apoptosis, post-transcriptional level, cell metabolism, and translation level, and does not affect the quality of recombinant protein. One miRNA may bind multiple target genes and regulate several metabolic pathways without increasing the burden of cell translation. The number of binding sites and the effect that may have on cells will surely be specific to the miRNA. However, the same miRNA can bind to multiple sites and regulate different metabolic pathways, which may have a superimposed effect if these pathways are beneficial for antibody production. If some pathways are favorable and some are unfavorable, this may offset some of the positive effects. There are many studies on the effect of a single miRNA on CHO cells. In the future, we can further explore the effect of the combined overexpression of two or more miRNAs on the yield and quality of recombinant proteins in CHO cells. It is also possible to study the effect of miRNA on the yield and quality of difficult-to-express proteins. The effect and mechanism of miRNA on the expression of recombinant protein in CHO cells still has much room for exploration.
